# Therapeutic enhancement and toxicity reduction of total glucosides of paeony combined with conventional therapy in juvenile idiopathic arthritis: a meta-analysis and network pharmacology

**DOI:** 10.3389/fped.2026.1937383

**Published:** 2026-10-28

**Authors:** Chao Yang, Guangqiang Ma, Yuling Yin, Zhengwei Xie, Lianjuan Yang, Yini Jiang, Yifei Rao

**Affiliations:** 1 School of Chinese and Western Integrative Medicine, Jiangxi University of Chinese Medicine, Nanchang, China; 2 School of Chinese Materia Medica, Bozhou University, Anhui, China

**Keywords:** enhancing efficacy and reducing toxicity, juvenile idiopathic arthritis, meta-analysis, network pharmacology, total glucosides of paeony

## Abstract

**Objective:**

This study aimed to evaluate the efficacy and safety of total glucosides of paeony (TGP) combined with conventional therapy in juvenile idiopathic arthritis (JIA) and explore the network regulatory mechanisms underlying TGP's synergistic enhancement and toxicity reduction effects.

**Method:**

We searched for randomized controlled trials (RCTs) on the efficacy of TGP combined with conventional therapy in JIA until 1 February 2026 (databases: PubMed, Embase, Web of Science, Cochrane Library, SinoMed, Chinese National Knowledge Infrastructure, Wanfang, and VIP). The outcome measures included overall effective rate, adverse reactions, inflammation indicators, autoantibody levels, and clinical symptoms. Subsequently, an integrative pharmacology approach was employed to construct a “drug target–efficacy/toxicity gene” interaction network, elucidating potential molecular mechanisms of TGP's synergistic and toxicity-reducing effects.

**Result:**

A total of 17 RCTs were included, involving 966 children with JIA. Compared with conventional Western medicine alone, TGP combination therapy significantly improved the overall response rate [risk ratio (RR) = 1.13, 95% confidence interval (CI) 1.07–1.19, *P* < 0.0001]and reduced the incidence of adverse events (RR = 0.63, 95% CI 0.43–0.91, *P* = 0.02), markedly ameliorating serum inflammatory indicators [interleukin (IL)-1, IL-17, tumor necrosis factor *α*, C-reactive protein, and erythrocyte sedimentation rate], autoantibody levels, and clinical symptoms in JIA patients. The “drug target–efficacy/toxicity gene” interaction network revealed that TGP exerts synergistic effects primarily by regulating energy metabolism, alleviating oxidative stress and inflammation, and suppressing synovitis and osteoclastogenesis. Its toxicity-reducing effects were mediated through the inhibition of hepatic inflammation, oxidative stress, and angiogenesis, as well as modulation of lipid and amino acid metabolism. Notably, oxidative stress and lipid metabolism emerged as potential common mechanisms for TGP enhancement and toxicity reduction.

**Conclusion:**

The therapy of TGP combined with conventional Western medicine could enhance clinical efficacy and reduce adverse events in JIA patients. The synergistic and toxicity-reducing mechanisms of TGP might be associated with multi-pathway regulation, particularly involving oxidative stress and lipid metabolism.

## 1 Introduction

Juvenile idiopathic arthritis (JIA) is the most common rheumatic immune disease in children, which is usually accompanied by damage to the eyes, liver, and multiple organs. It results in serious physical and psychological trauma to children because of its chronic, recurrent, and disabling nature. Currently, the treatment regimen for JIA is mainly based on Western medicine, including non-steroidal anti-inflammatory drugs (NSAIDs), disease-modifying antirheumatic drugs (DMARDs), glucocorticoids (GCs), and biological agents (1, 2). Follow-up studies have shown that some children with JIA still have poor medication compliance and generally poor efficacy after treatment with conventional Western medicine (3, 4). In addition, conventional Western drug treatments such as NSAIDs and DMARDs often cause various adverse reactions, including dizziness, rash, gastrointestinal reactions, and multi-organ toxicity (particularly liver toxicity) (5, 6). In recent years, an increasing number of clinical and experimental studies have shown that the combined use of traditional Chinese medicine and conventional Western medicine can improve therapeutic efficacy while reducing toxicity, further ensuring effectiveness and safety (7, 8).

Total glucosides of paeony (TGP) is the active component extracted from the dried roots of *Paeonia lactiflora* Pall. It primarily consists of paeoniflorin, hydroxypaeoniflorin, paeoniflorin glycoside, paeoniflorin lactone glycoside, and benzyl paeoniflorin glycoside. Among these, paeoniflorin is the main active ingredient of TGP, accounting for more than 90% (9). Clinically, TGP’s primary indication is rheumatoid arthritis. It can exert significant anti-inflammatory and immunomodulatory effects through multiple pathways, such as inhibiting the activation of immune cells and synovial cells and reducing the production of inflammatory substances (10). In recent years, TGP has also been used in the treatment of JIA due to its low side effects, good safety profile, and outstanding efficacy (11). However, there are currently few reports on the therapeutic enhancement and toxicity reduction of TGP combined with Western medicine for JIA, and its potential mechanisms remain unclear. In this study, we conducted a meta-analysis of TGP combined with conventional Western medicine therapy in JIA, comprehensively evaluating overall efficacy, adverse reactions, inflammatory response indicators, autoantibody levels, and clinical symptom indicators. In addition, the “drug target–efficacy/toxicity gene” interaction network was constructed through network pharmacology analysis. These results will provide evidence and data support for guiding the rational use of TGP in the clinical treatment of JIA.

## 2 Methods

### 2.1 The meta-analysis of the combination of TGP and conventional Western medicine against JIA

#### 2.1.1 Search strategies

We searched PubMed, Embase, Web of Science, Cochrane Library, SinoMed, Chinese National Knowledge Infrastructure, Wanfang, and VIP databases from inception to 1 February 2026. The initial search items were “Total glucosides of paeony” OR “TGP”, “juvenile idiopathic arthritis” OR “JIA”, Randomized controlled trial OR Controlled trial OR Clinical observation. The base search formula was #1 (Total glucosides of paeony OR TGP) AND #2 (juvenile idiopathic arthritis OR JIA) AND#3 (Randomized controlled trial OR Controlled trial OR Clinical observation).

#### 2.1.2 Eligibility criteria

The inclusion criteria were (1) patients met the JIA classification criteria proposed and revised by the International League of Associations for Rheumatology (ILAR) in 2001 (12); (2) RCTs of TGP treatment for JIA; (3) interventions: the experimental group used a combination of TGP and conventional Western medicine to treat JIA, while the control groups used only conventional Western medicine; (4) outcomes: overall effective rate, adverse reactions, inflammatory response indicators, autoantibody levels, and clinical symptoms; (5) the literature was limited to English or Chinese.

The exclusion criteria were (1) interventions included self-made TGP preparations (including tinctures, wines, and syrups), Chinese herbal medicines, or compounds containing TGP; (2) patients had other serious diseases; (3) animal experiments, literature reviews, and conference abstracts; (4) repetitively published studies; (5) studies with incomplete or incorrect data.

#### 2.1.3 Data extraction

Two investigators independently assessed all trials for eligibility and extracted data by screening the titles and abstracts. If disagreements occurred, a third investigator resolved them by retrieving the full articles and discussing the findings. Data extracted included (1) basic information, including title, source, author, and year; (2) baseline characteristics of the study population, including the number of trial participants, age, gender, sample size, and diagnostic criteria; (3) intervention details included study group, control group, drug, dosage, and cycle; (4) data on outcome indicators and measures of overall effective rate, adverse reactions, inflammatory response indicators, autoantibody levels, and clinical symptoms.

#### 2.1.4 Bias risk assessment

We considered the following aspects for the risk of bias assessment: random sequence generation, allocation concealment, blinding of participants and personnel, blinding of outcome assessment, incomplete outcome data, selective reporting, and other sources of bias. Risk of bias assessment was performed using Review Manager (version 5.4, The Cochrane Collaboration, London, United Kingdom). Each aspect of the risk of bias in each RCT was assessed as “low risk,” “high risk,” or “unclear risk.”

#### 2.1.5 Statistical analysis

We applied Review Manager 5.4 software to perform the meta-analyses. The normalized mean difference (MD) was calculated for continuous variables, while the risk ratios (RRs) were used as the effect analysis statistic for dichotomous variables. All results were provided with 95% confidence intervals (95% CIs). *I^2^* statistics and chi-square tests were used to assess statistical heterogeneity. Fixed effects were used if there was no heterogeneity between studies (*I^2^* < 50%, *P* > 0.05). If there was heterogeneity (*I^2^* > 50%, *P* ≤ 0.05), the source of heterogeneity was analyzed, and a meta-analysis was performed with random effects after excluding the influence of heterogeneity. For outcomes with *I^2^* > 80%, leave-one-out sensitivity analyses were further performed by sequentially removing each study and recalculating the pooled estimate. If more than 10 studies tested the same result, a funnel plot was used to assess publication bias. All data results were considered statistically significant at *P* < 0.05.

### 2.2 The network pharmacology analysis of therapeutic enhancement and toxicity reduction of TGP combined with conventional therapy in JIA

#### 2.2.1 Screening of TGP-related targets

The related targets of TGP were obtained from the TCMIP v2.0 (Integrative Pharmacology-based Research Platform of Traditional Chinese Medicine, version 2.0, http://www.tcmip.cn/TCMIP/index.php/). We screened 300 TGP-related targets, as shown in the Supplementary Material.

#### 2.2.2 Acquisition of genes related to JIA and drug-induced liver injury

JIA-related genes were obtained from the GEO database (http://www.ncbi.nlm.nih.gov/geo/). We acquired peripheral blood cell and synovial tissue transcriptomic data from JIA patients and normal controls (GEO Accession Numbers: GSE17755 and GSE71595). Then, the differential expression analysis tool GEO2R was used to identify differentially expressed genes between JIA patients and healthy controls. Furthermore, drug-induced liver injury-related genes were obtained from the DisGeNET database (http://www.disgenet.org/), DrugBank database (https://www.drugbank.com), and research literature, collecting 113, 57, and 802 genes, respectively. After removing repetitions, we obtained 582 genes associated with drug-induced liver injury, as shown in the Supplementary Material.

#### 2.2.3 Construction of “drug target–efficacy/toxicity gene” interaction network

To further identify the core regulatory genes, the “drug target–efficacy/toxicity gene” interaction network was constructed using the STRING database (https://string-db.org/) with the “association network mining” analysis module. We screened key network target genes by calculating three network topological characteristics (connectivity, betweenness, and compactness). Subsequently, pathway enrichment analysis was performed using the Reactome database (https://reactome.org) to screen for molecular pathways significantly involved in the key network target genes. Finally, Cytoscape 3.7.2 software was used for network visualization.

## 3 Results

### 3.1 The meta-analysis of the combination of TGP and conventional Western medicine against JIA

We searched a total of 252 articles, including 221 in Chinese and 31 in English. After excluding 190 duplicate studies and 45 reviews, conferences, and case studies, 17 published studies were available for the meta-analysis after reading the titles, abstracts, and full texts (Figure 1).

**Figure 1:**
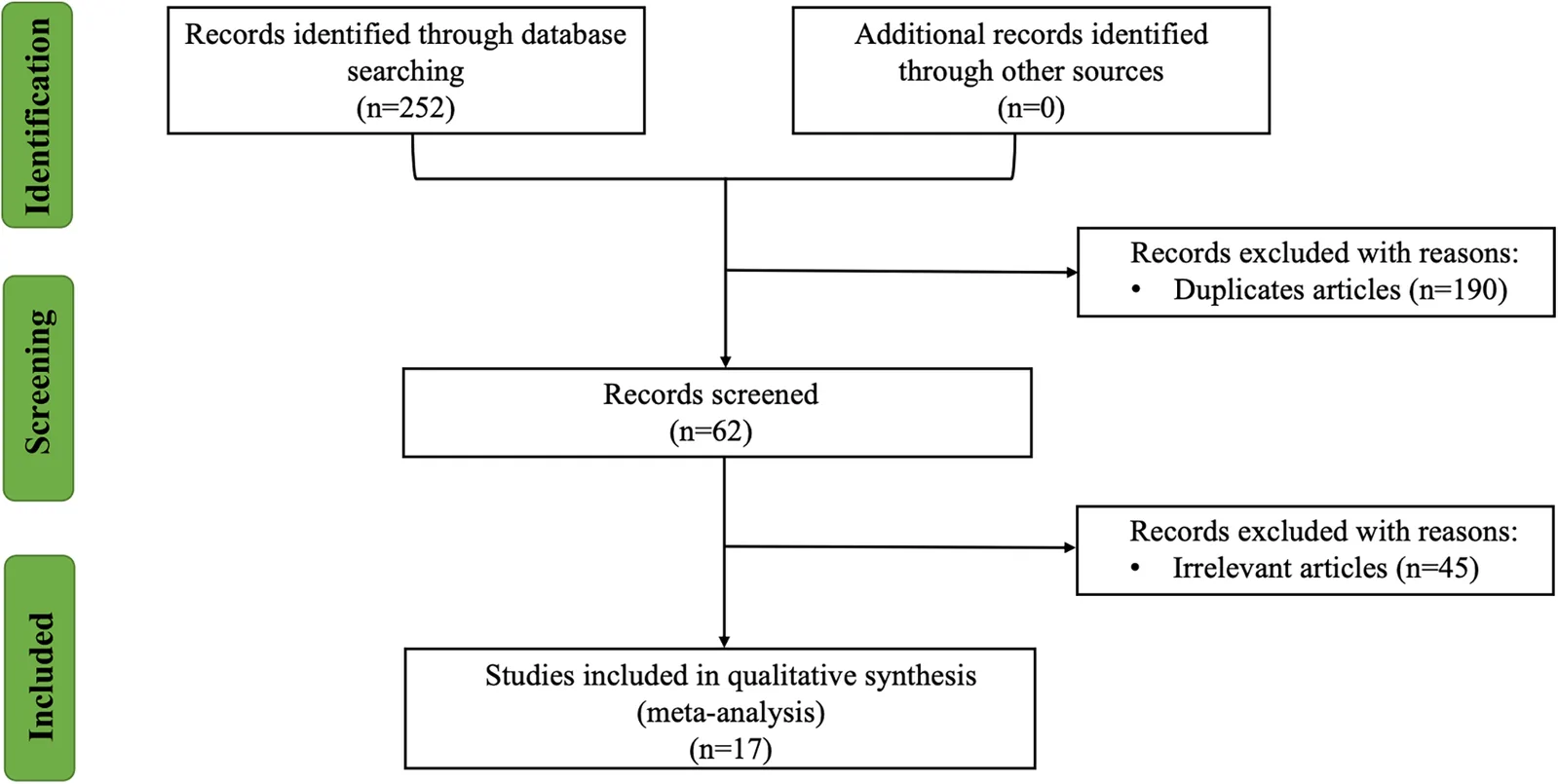
Flow diagram of all studies included in this meta-analysis; of 252 studies identified via electronic and manual search, a total of 17 studies were included.

#### 3.1.1 Study characteristics

We selected conventional Western medicine as the control to compare the effects of conventional Western medicine + TGP interventions on JIA. A total of 966 patients were included, with a minimum sample size of 36 and a maximum sample size of 94 in the study. The treatment doses of TGP were as follows: 30 mg·day·kg^−1^, 30–60 mg·day·kg^−1^, 1.8 g·day^−1^, 3–6 mg·day·kg^−1^, 1.2 g·day^−1^, and 0.03–0.06 mg·day·kg^−1^, with a treatment duration ranging from 12 weeks to 6 months. Detailed information is presented in Table 1.

**Table 1:** Basic characteristics of included studies.

Author (year)	Sample size		Intervention measures		TGP dosage	Follow-up duration
	C	T	C	T		
Hang et al. (2024) (13)	44	44	MTX	TGP + MTX	30 mg·day·kg^−1^	6 months
Zhu et al. (2024) (14)	40	40	NSAIDs + DMARDs	TGP + NSAIDs + DMARDs	1.8 g·day^−1^	3 months
Liu et al. (2020) (15)	46	46	NSAIDs + GCs	TGP + NSAIDs + GCs	30 mg·day·kg^−1^	6 months
Liu et al. (2020) (16)	23	23	rhTNFR	TGP + rhTNFR	30 mg·day·kg^−1^	3 months
Wei et al. (2019) (17)	30	30	NSAIDs + DMARDs	TGP + NSAIDs + DMARDs	30 mg·day·kg^−1^	6 months
Liu et al. (2018) (18)	47	47	DMARDs	TGP + DMARDs	30 mg·day·kg^−1^	6 months
Li et al. (2017) (19)	28	28	DMARDs	TGP + DMARDs	30 mg·day·kg^−1^	12 weeks
Yue et al. (2017) (20)	20	20	DMARDs	TGP + DMARDs	30 mg·day·kg^−1^	6 months
Li et al. (2016) (21)	45	45	NSAIDs + MTX	TGP + NSAIDs + MTX	30–60 mg·day·kg^−1^	6 months
Quan and Jin (2015) (22)	40	40	NA	TGP + NA	30 mg·day·kg^−1^	6 months
Wang (2012) (23)	30	31	DMARDs	TGP + DMARDs	1.8 g·day^−1^	12 weeks
Sun (2011) (24)	26	25	DMARDs	TGP + DMARDs	3–6 mg·day·kg^−1^	26 weeks
Tong and Shui (2009) (25)	42	42	NSAIDs + DMARDs	TGP + NSAIDs + DMARDs	3–6 mg·day·kg^−1^	6 months
Li et al. (2008) (26)	18	18	NSAIDs + DMARDs	TGP + NSAIDs + DMARDs	30 mg·day·kg^−1^	12 weeks
Liu (2008) (27)	35	35	NSAIDs + DMARDs	TGP + NSAIDs + DMARDs	NA	6 months
Liu et al. (2007) (28)	20	20	DMARDs	TGP + DMARDs	1.2 g·day^−1^	6 months
Zang (2004) (29)	26	42	DMARDs	TGP + DMARDs	0.03–0.06 mg·day·kg^−1^	6 months

NA, data not available; T, treatment group; C, control group; MTX, methotrexate; NSAIDs, non-steroidal anti-inflammatory drugs; GC, glucocorticoid; rhTNFR, recombinant human TNF receptor.

#### 3.1.2 Bias risk assessment

Two investigators independently assessed the risk of bias in the included studies and cross-checked the results. Data were collated from 17 published articles. Seven RCTs (16–18, 20–23) were assigned to random groups using the “random number table” method or the “computer random sequence generation method,” while the remaining studies failed to specify the randomization method. None of the studies reported allocation concealment, double-blinding, participant dropouts and their causes, or blinding of outcome assessments. Regarding data completeness, selective reporting, and other aspects, a low risk of bias was observed.

#### 3.1.3 Outcome of overall effective rate

Twelve studies involving 801 patients reported the overall response rate. Heterogeneity analysis showed low heterogeneity among studies (*I*^2^ = 48%, *P* = 0.03), and the fixed-effect model was used. Compared with conventional Western medicine therapy alone, TGP combination therapy significantly improved the overall response rate (RR = 1.13, 95% CI 1.07–1.19, *P* < 0.0001), as shown in Table 2.

**Table 2:** The details of outcome measures.

Outcome measures	Number of RCTs	Sample size (C)	Sample size (T)	*I^2^* (%)	MD/RR (95% CI)	*P*
Overall effective rate	12	392	409	48	1.13 (1.07 to 1.19)	<0.0001
Adverse reactions	8	251	269	0	0.63 (0.43 to 0.91)	0.02
IL-1	3	94	94	63	−8.30 (−12.05 to −4.54)	<0.0001
IL-17	3	93	93	96	−5.58 (−10.13 to −1.03)	0.02
TNF-α	7	232	232	99	−29.62 (−43.27 to −15.96)	<0.0001
CRP	11	341	359	89	−3.30 (−4.63 to −1.96)	<0.00001
ESR	13	409	427	84	−5.96 (−8.03 to −3.88)	<0.00001
RF	4	118	119	98	−8.74 (−18.54 to 1.05)	0.08
Morning stiffness	9	284	302	99	−4.59 (−6.10 to −3.07)	<0.00001
Joint pain index	8	242	260	99	−3.96 (−5.24 to −2.67)	<0.00001
Joint swelling index	7	213	215	99	−4.35 (−7.57 to −1.14)	0.008

T, treatment group; C, control group.

#### 3.1.4 Outcome of adverse reactions

Eight studies involving 520 patients reported related adverse reactions. The results showed that the adverse reactions associated with conventional Western medicine for JIA primarily manifested as hepatotoxicity, gastrointestinal toxicity, hematological toxicity, and reproductive toxicity. Among these, the incidence of liver toxicity was the highest, with abnormal liver function being the most common. Heterogeneity analysis showed low heterogeneity among studies (*I*^2^ = 0%, *P* = 0.77), and the fixed-effect model was used. Compared with conventional Western medicine therapy alone, TGP combination therapy significantly reduced the incidence of adverse reactions (RR = 0.63, 95% CI 0.43–0.91, *P* = 0.02). These results support the idea that TGP combination therapy could alleviate the adverse reactions caused by conventional Western medicine, as shown in Table 2.

#### 3.1.5 Outcome of inflammation indicator levels

Three studies involving 188 patients reported serum interleukin-1 (IL-1) levels. Heterogeneity analysis revealed significant heterogeneity among the studies (*I*^2^ = 63%, *P* = 0.07), and the random-effects model was used. Compared with conventional Western medicine therapy alone, TGP combination therapy significantly reduced serum IL-1 levels (MD = −8.30, 95% CI −12.05 to −4.54, *P* < 0.0001), as shown in Table 2.

In total, 3 studies involving 186 patients reported serum interleukin-17 (IL-17) levels. Heterogeneity analysis revealed significant heterogeneity among the studies (*I*^2^ = 96%, *P* < 0.00001), and the random-effects model was used. Compared with conventional Western medicine therapy alone, TGP combination therapy significantly reduced serum IL-17 levels (MD = −5.58, 95% CI −10.13 to −1.03, *P* = 0.02),as shown in Table 2.

Seven studies involving 464 patients reported serum tumor necrosis factor *α* (TNF-α) levels. Heterogeneity analysis revealed significant heterogeneity among the studies (*I*^2^ = 99%, *P* < 0.00001), and the random-effects model was used. Compared with conventional Western medicine therapy alone, TGP combination therapy significantly reduced serum TNF-α levels (MD = −29.62, 95% CI −43.27 to −15.96, *P* < 0.0001), as shown in Table 2.

Eleven studies involving 700 patients reported serum C-reactive protein (CRP) levels. Heterogeneity analysis revealed significant heterogeneity among the studies (*I*^2^ = 89%, *P* < 0.00001), and the random-effects model was used. Compared with conventional Western medicine therapy alone, TGP combination therapy significantly reduced serum CRP levels (MD = −3.30, 95% CI −4.63 to −1.96, *P* < 0.00001), as shown in Table 2.

Thirteen studies involving 836 patients reported erythrocyte sedimentation rate (ESR) levels. Heterogeneity analysis revealed significant heterogeneity among the studies (*I*^2^ = 84%, *P* < 0.00001), and the random-effects model was used. Compared with conventional Western medicine therapy alone, TGP combination therapy significantly reduced ESR levels (MD = −5.96, 95% CI −8.03 to −3.88, *P* < 0.00001), as shown in Table 2.

#### 3.1.6 Outcome of autoantibody levels

Four studies involving 237 patients reported rheumatoid factor (RF) levels. Heterogeneity analysis revealed significant heterogeneity among the studies (*I*^2^ = 98%, *P* < 0.00001), and the random-effects model was used. Compared with conventional Western medicine therapy alone, TGP combination therapy showed a downward trend in RF levels (MD = −8.74, 95% CI −18.54 to 1.05, *P* = 0.08), as shown in Table 2.

#### 3.1.7 Outcome of clinical symptoms

Nine studies involving 586 patients reported the duration of morning stiffness. Heterogeneity analysis revealed significant heterogeneity among the studies (*I*^2^ = 99%, *P* < 0.00001), and the random-effects model was used. Compared with conventional Western medicine therapy alone, TGP combination therapy showed a statistically significant improvement in morning stiffness duration (MD = −4.59, 95% CI −6.10 to −3.07, *P* < 0.00001), as shown in Table 2.

Eight studies involving 502 patients reported the joint pain index. Heterogeneity analysis revealed significant heterogeneity among the studies (*I*^2^ = 99%, *P* < 0.00001), and the random-effects model was used. Compared with conventional Western medicine therapy alone, TGP combination therapy significantly reduced the joint pain index (MD = −3.96, 95% CI −5.24 to −2.67, *P* < 0.00001), as shown in Table 2.

Seven studies involving 428 patients reported the joint swelling index. Heterogeneity analysis revealed significant heterogeneity among the studies (*I^2^* = 99%, *P* < 0.00001), and the random-effects model was used. Compared with conventional Western medicine therapy alone, TGP combination therapy significantly reduced the joint swelling index (MD = −4.35, 95% CI −7.57 to −1.14, *P* = 0.008), as shown in Table 2.

### 3.2 The network pharmacology analysis of therapeutic enhancement and toxicity reduction of TGP combined with conventional therapy in JIA

#### 3.2.1 Exploring the biological implications of core network targets for enhancing efficacy

Numerous studies have shown that the occurrence and development of JIA are closely related to the involvement of blood cells such as red blood cells, white blood cells, and platelets (1). In this study, we further constructed a differential gene–protein interaction network between TGP targets and peripheral blood cells in JIA patients, suggesting that the core network targets of TGP therapy for JIA primarily achieve synergistic effects by regulating blood cell energy metabolism, improving metabolic disorders and immune imbalance, alleviating oxidative stress, and reducing systemic inflammatory responses in the hematopoietic system, as shown in Figure 2. Furthermore, as the main pathological link in JIA, we also constructed a protein interaction network of differentially expressed genes between TGP targets and synovial tissue from JIA patients, and found that the core network targets of TGP treatment for JIA mainly achieve synergistic effects by inhibiting synovial inflammation and osteoclast generation, regulating synovial chemokines, inhibiting the production of pathogenic autoantibodies, regulating growth hormone and amino acid synthesis, and improving synovial cell lipid metabolism disorders, as shown in Figure 3.

**Figure 2:**
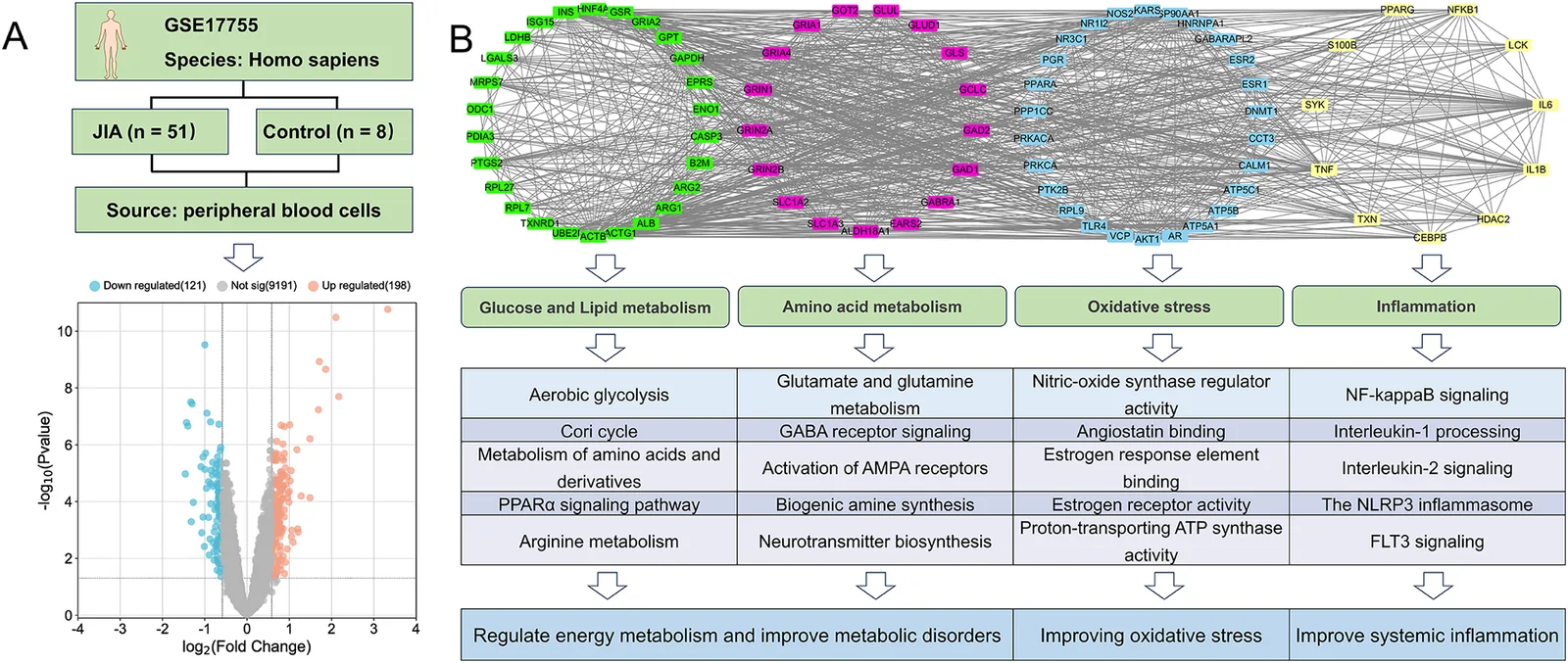
Protein interaction network of differentially expressed genes between TGP targets and JIA patients in peripheral blood cells. **(A)** Gene acquisition process for identifying differences in peripheral blood cells between JIA patients and healthy controls. **(B)** Protein–protein interaction network and kyoto encyclopedia of genes and genomes (KEGG) enrichment analysis between differentially expressed genes from JIA patients’ peripheral blood cells and TGP targets.

**Figure 3:**
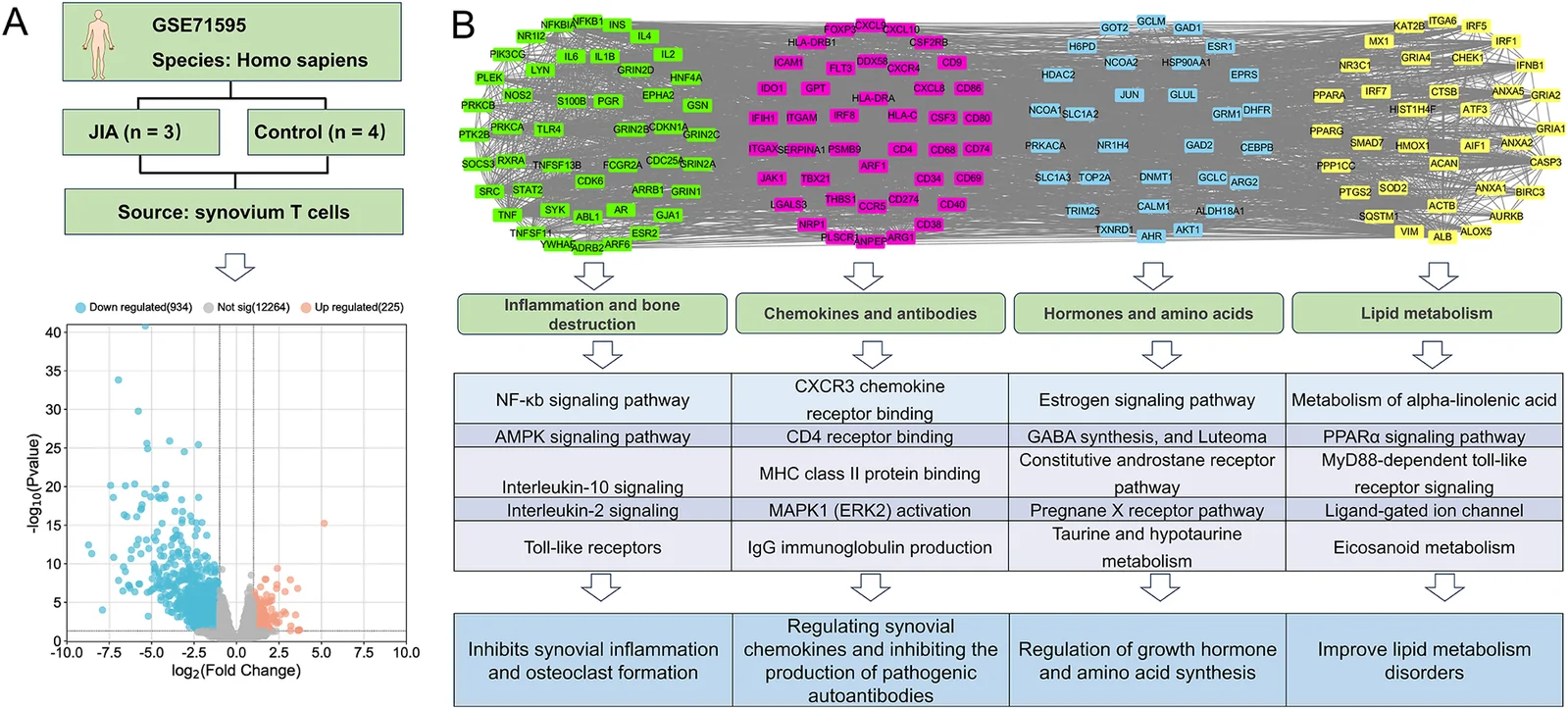
Protein interaction network of differentially expressed genes between TGP targets and JIA patients in synovial tissue. **(A)** Gene acquisition process for differences in synovial tissue between JIA patients and healthy controls. **(B)** Protein–protein interaction network and KEGG enrichment analysis between differentially expressed genes from JIA patients’ synovial tissue and TGP targets.

#### 3.2.2 Exploring the biological implications of core network targets for reducing toxicity

Researchers have reported that conventional Western medications often cause drug-induced liver damage during the treatment of JIA, such as NSAIDs causing liver hypersensitivity reactions, leading to abnormal liver metabolism, and cumulatively inducing liver toxicity. Generally, hepatotoxicity is characterized by poor predictability, high incidence, and rapid progression, which is also an important reason for the limited clinical application of NSAIDs. In addition, MTX and leflunomide can cause elevated serum transaminase levels, and long-term use might lead to fatty liver, liver fibrosis, or even cirrhosis. Therefore, this study aimed to explore the core network targets for TGP against drug-induced liver injury by constructing a “drug target–toxicity gene” interaction network. The results showed that TGP primarily achieves its detoxification effects by reducing hepatic microenvironment inflammation, regulating oxidative stress and angiogenesis, inhibiting hepatocyte apoptosis, and improving lipid metabolism and amino acid metabolism, as shown in Figure 4.

**Figure 4:**
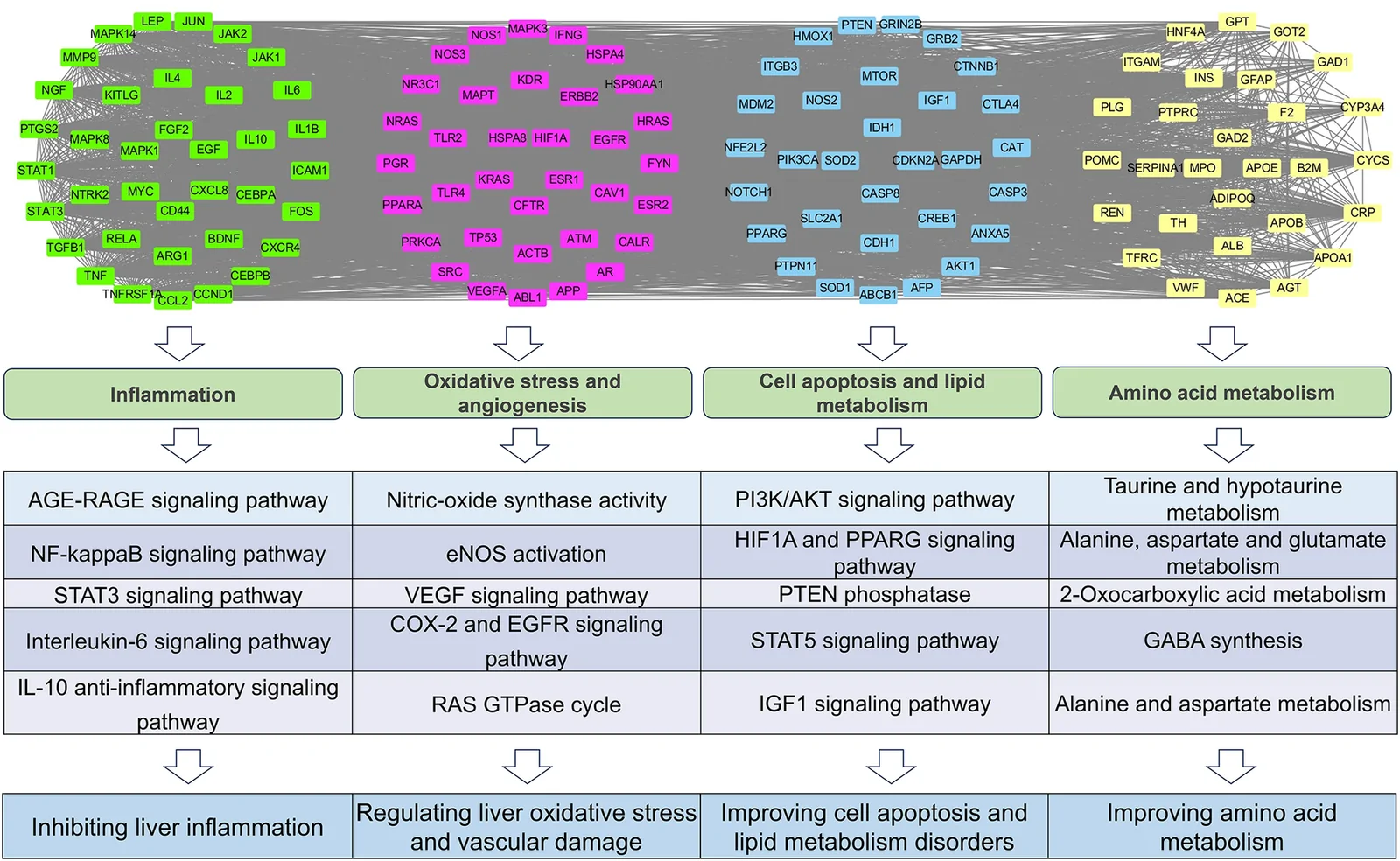
TGP target and drug-induced liver toxicity core gene protein interaction network.

## 4 Discussion

NSAIDs and traditional antirheumatic drugs are widely used in the treatment of JIA. Among them, NSAIDs exert anti-inflammatory and analgesic effects by inhibiting cyclooxygenase, thereby reducing the production of prostaglandins (PGs). Traditional antirheumatic drugs mainly inhibit lymphocyte function and reduce the secretion of pro-inflammatory cytokines, thereby suppressing the immune system's attack on its own organs and tissues, ultimately achieving therapeutic effects for JIA (30). However, existing research indicates that conventional Western medicines still have limitations in clinical application, such as poor drug compliance and mediocre efficacy. Furthermore, the clinical application of conventional Western medicines can induce a variety of adverse reactions, with liver toxicity being particularly prominent, which greatly limits their use in pediatric patients (3). Therefore, in the clinical use of conventional drugs for the treatment of JIA, it is necessary to combine other drugs while ensuring efficacy to reduce adverse reactions. Research has shown that Chinese herbal medicine combinations are based on the theory of syndrome differentiation and treatment in traditional Chinese medicine, which involves the rational combination of two or more drugs to achieve synergistic effects, thereby reducing toxicity, enhancing efficacy, and moderating drug properties. To address liver toxicity, herbs such as *Paeonia lactiflora* Pall. are often combined to counteract damage to liver and kidney function while ensuring therapeutic efficacy (31). In the “Guidelines for the Diagnosis and Treatment of Rheumatoid Arthritis” (32), TGP is always recommended to be used in combination with conventional Western medicine for the treatment of various types of rheumatoid arthritis.

This study included 17 RCTs evaluating the efficacy of TGP combined with conventional Western medicine for JIA. The results showed that the combination of TGP significantly increased the overall efficacy rate and reduced the incidence of adverse reactions, improved serum inflammatory response indicators and autoantibody levels in JIA patients, and effectively alleviated clinical symptoms. These findings suggest that TGP combined with conventional therapy might be more effective and reduce adverse reactions in children with JIA. Despite the high heterogeneity observed for several outcomes (*I*^2^ > 80%), leave-one-out sensitivity analyses showed that the pooled estimates remained stable and the overall conclusions were not materially altered. Based on the results of the meta-analysis, we further explored the network regulatory mechanisms underlying the synergistic and toxicity-reducing effects of TGP in JIA. The results suggested that TGP may exert its synergistic effect by regulating energy metabolism, improving oxidative stress and inflammation, and inhibiting synovial inflammation and osteoclast formation. In terms of toxicity reduction, TGP inhibits liver inflammation, oxidative stress, and vascular proliferation, while also regulating lipid and amino acid metabolism. Among these, oxidative stress and lipid metabolism may be common mechanisms for TGP's synergistic detoxification, and pathway enrichment analysis indicated that nitric oxide synthase (iNOS) activity and the peroxisome proliferator-activated receptor (PPAR) signaling pathway are shared pathways for synergistic detoxification. Numerous studies have shown a regulatory relationship between iNOS and PPAR. Activating PPAR could inhibit iNOS and reduce nitric oxide (NO) production. Both pathways are related to lipid oxidation and synthesis (33, 34).

Regarding therapeutic enhancement, TGP might activate the PPAR signaling pathway to improve inflammation, cartilage damage, and energy metabolism, which, combined with conventional Western medicine, could enhance clinical efficacy. Previous studies have shown that PPAR is negatively correlated with disease severity in multiple animal models of arthritis, and PPAR agonists could improve arthritis symptoms (35). Similarly, cartilage destruction is one of the main pathological manifestations of JIA. Research has reported that spontaneous cartilage destruction occurs in mice after specific knockout of PPARγ, further suggesting that PPARγ is an important regulatory factor in cartilage damage (36). The activation of PPARγ can directly regulate multiple antioxidant genes during oxidative stress. Studies have demonstrated that PPARγ agonists could activate the main antioxidant stress regulatory pathway Nrf2/HO-1 (37) and inhibit the levels of genes such as iNOS, SOD2, and Col2a1 in chondrocytes (38), which are closely related to cartilage destruction and synovial hyperplasia. In addition, PPARγ can also inhibit inflammatory response genes, such as IL-2, IL-6, IL-8, TNF, and metalloproteinases. Several studies have shown that PPARβ/δ also regulates lipid metabolism by promoting fatty acid oxidation, lipoprotein metabolism, glucose utilization, and anti-inflammatory effects (39, 40).

In terms of toxicity reduction, research has shown that both NSAIDs and DMARDs can disrupt the balance of reactive oxygen species (ROS) metabolism in the liver, causing the liver to remain in a state of oxidative stress for long periods, ultimately leading to liver damage (41, 42). iNOS is involved in oxidative stress damage to cells; mitochondria are the primary site for the production of ROS, and the dysregulation of iNOS in mitochondria can lead to excessive production of ROS, resulting in oxidative stress and mitochondrial dysfunction, ultimately causing cell apoptosis (43). Under oxidative stress conditions, ROS attack cell membranes, producing reactive intermediates that activate a chain reaction of proliferation, forming a series of lipid free radicals and degradation products—malondialdehyde (MDA). This process is known as lipid peroxidation. Lipid peroxidation not only directly damages membrane phospholipids, but its peroxidation chain reaction products also have high biological activity, which can destroy the biological activity of DNA, proteins, and enzymes, and can serve as cell death signals to induce programmed cell death (44). Activation of the PPAR signaling pathway can reduce ROS production, thereby alleviating oxidative stress. Furthermore, PPAR activation can improve mitochondrial damage by inhibiting mitochondrial vacuolation (45). Recent studies also show that it can exert hepatoprotective effects by modulating M2 macrophage polarization (46). Based on the above results, TGP might alleviate drug-induced liver toxicity by regulating the PPAR-iNOS signaling axis.

TGP is a representative product of integrating traditional Chinese medicine with modern medical technology. Recent research emphasizes that integrating traditional, complementary, and alternative medicine with modern biomedicine requires rigorous evidence-based evaluation and standardized quality control, while also facing challenges such as methodological heterogeneity and variable evidence quality (47). The integration of meta-analysis with network pharmacology has become an important paradigm for studying natural products in rheumatic diseases; for example, a recent meta-analysis of placebo-controlled RCTs confirmed the clinical efficacy of curcumin in rheumatoid arthritis while highlighting limitations such as small sample sizes and very low certainty of evidence (48). The present study adopted a similar integrative approach to explore the synergistic and toxicity-reducing mechanisms of TGP in JIA.

In summary, TGP combined with conventional therapy can effectively enhance therapeutic efficacy and reduce adverse reactions in children with JIA. However, due to insufficient reporting of randomization, allocation concealment, and blinding in many included RCTs, the reliability of the pooled results is limited, and definitive conclusions cannot be drawn. Network pharmacology analysis further suggests that the synergistic and detoxifying mechanisms of TGP may involve the regulation of oxidative stress and lipid metabolism, particularly the PPAR-iNOS signaling axis. These findings help explain the mechanism by which TGP combined with conventional therapy enhances efficacy and reduces toxicity in JIA, and provide a reference for clinicians and researchers. Further high-quality RCTs with rigorous methodology and experimental validation are needed to confirm these findings.

## 5 Limitations

Several limitations should be acknowledged. The included RCTs had incomplete reporting of randomization, allocation concealment, and blinding, along with high heterogeneity and small sample sizes (minimum 36 patients), which may limit the reliability of pooled estimates. Only Chinese- and English-language literature was searched, and most included studies were sourced from Chinese databases. Funnel plots for outcomes with >10 studies were symmetrical, suggesting no obvious publication bias; however, publication bias could not be fully assessed for outcomes with fewer than 10 studies. The network pharmacology results are hypothesis-generating, lack experimental validation, and rely on relatively small GEO datasets. Despite these limitations, the findings provide a useful basis for future research.

## Data Availability

The datasets presented in this study can be found in online repositories. The names of the repository/repositories and accession number(s) can be found in the article/Supplementary Material.
