# Supplementary material for: Therapeutic enhancement and toxicity reduction of total glucosides of paeony combined with conventional therapy in juvenile idiopathic arthritis: a meta-analysis and network pharmacology
Source: Front Pediatr. 2026 Oct 28;14:1937383. doi: 10.3389/fped.2026.1937383 (PMC13752100; doi:10.3389/fped.2026.1937383)
Supplement: Supplementary file 1 [file Supplementaryfile1.docx]

**Supplementary Material.**

1. The related targets of total glucosides of paeony (TGP) from the TCMIP v2.0.
2. The transcriptomic data from juvenile idiopathic arthritis (JIA) patients and normal controls in peripheral blood cell (GEO Accession Number: GSE17755).
3. The transcriptomic data from juvenile idiopathic arthritis (JIA) patients and normal controls in synovial tissue (GEO Accession Number: GSE71595).
4. Drug-induced liver injury-related genes from the DisGeNET database, DrugBank database and research literature.
5. Supplementary Figure S1. Funnel plot of publication bias for the overall effective rate.
6. Supplementary Figure S2. Funnel plot of publication bias for c-reactive protein (CRP).
7. Supplementary Figure S3. Funnel plot of publication bias for erythrocyte sedimentation rate (ESR).

| 1. TGP-related targets | 2. JIA-related genes   (GSE17755) | 3. JIA-related genes   (GSE71595) | 4. Drug-induced liver injury-related genes |
| --- | --- | --- | --- |
| AKR1C1  AKR1C2  AR  CLEC4E  ESR1  ESR2  GABRA1  GABRA2  GABRA3  GABRA4  GABRA5  GABRA6  GABRB1  GABRB2  GABRB3  GABRD  GABRE  GABRG1  GABRG2  GABRG3  GABRP  GABRQ  GRIN1  GRIN2A  GRIN2B  GRIN2C  GRIN2D  GRIN3A  GRIN3B  HSD17B1  LIP3  LSS  NCOA2  NR1I2  NR1I3  NR3C1  NR3C2  PGR  PPARA  RORA  SIGMAR1  SULT2A1  SULT2B1  VDR  CA2  CTSB  DAO  HRSP12  PRDX5  RAB9A  SLC15A1  ANXA1  ATP1A1  ATP1A2  ATP1A3  CASP3  GLRA3  HSD11B1  HSD11B2  HSD3B1  LPL  NFKB1  NFKB2  PPP1CC  SLCO1B3  TNF  YWHAE  ACTB  AHR  AKT1  ATP5A1  ATP5B  ATP5C1  CBR1  CDK6  CEBPB  COMT  CSNK2A1  CSNK2B  CYP1B1  DHFRL1  DNMT1  EIF3F  ESRRA  ESRRB  GPER1  HCK  HIBCH  HSP90AA1  HSPA2  JAK1  NCOA1  NQO2  PIK3CG  PIM1  PTK2B  RUVBL2  SF3B3  SHBG  STK17B  TOP2A  UBA1  UGT3A1  CTRB1  LCN2  MIF  PTGS1  PTGS2  TYR  AKR1C3  ALDH2  AURKB  CA1  CA12  CA14  CA3  CA4  CA5A  CA5B  CA6  CA7  CA9  KCNMA1  PRKACA  PRKCA  PRKCB  S100P  SQLE  SYK  AMY1A  AMY2A  AMY2B  BAMF_RS28815  GCK  GLT6D1  GLTP  GNPDA1  HK1  IFNB1  KRTAP5-2  KRTAP5-3  LCT  LCTL  LGALS2  LGALS3  LGALS7  MB  MBL2  NCAN  NUDT9  PYGL  PYGM  SFTPD  SIGLEC1  SMARCA5  TM0024  aroQ  ABL1  ADH5  ARF1  ARF6  C8G  CALM1  CYP2C8  ECI2  FKBP1A  FURIN  GM2A  GUCA1A  HNF4A  HNF4G  INS  LALBA  LTF  LY96  NR1H4  PAEP  PKIA  PLA2G1B  PLA2G2A  PLA2G2D  PLA2G2E  PMP2  PPARD  PPARG  PPA_RS05235  PPP3CA  PPP3R1  PPT1  PVR  RCVRN  RHO  RXRA  S100B  SEC14L2  TLR4  TM1468  TRAPPC3  VLDLR  ALOX5  HDAC2  HOXA10  IGHG2  NOS2  NPPB  NR0B1  PRLR  ABO  ALB  E  ADRB1  ADRB2  AMD1  CASR  DR_A0149  ODC1  SMOX  SMS  TXNRD1  AADAT  AASS  ABAT  ALDH18A1  ARG1  ARG2  ASNS  BCAT1  BCAT2  CCBL2  CPQ  DNPEP  EARS2  ENPEP  EPRS  FOLH1  FPGS  FTCD  GAD1  GAD2  GATB  GATM  GCLC  GCLM  GGCX  GIG18  GLS  GLS2  GLUD1  GLUD2  GLUL  GMPS  GOT1  GOT2  GPT  GPT2  GRIA1  GRIA2  GRIA3  GRIA4  GRID1  GRID2  GRIK1  GRIK2  GRIK3  GRIK4  GRIK5  GRM1  GRM4  GRM7  GRM8  LGSN  NADSYN1  NAGS  OAT  OAZ1  OAZ2  OAZ3  OPLAH  OTC  PFAS  PSAT1  SLC1A1  SLC1A2  SLC1A3  SLC1A6  SLC1A7  SLC25A15  SLC25A18  SLC25A2  SLC25A22  SLC7A1  SLC7A11  SLC7A2  SLC7A3  SLC7A4  TAT  TM0268  AADACL2  IL1B  IL6 | IL31  RNA5-8SN1  RNA5-8SN5  RNA5-8SN3  RNA5-8SN4  RNA5-8SN2  ZCCHC12  IL5  IGHM  LOC112268313  LRRN3  AIF1  GPR15  EDN1  LOC728989  AEBP1  CNKSR2  HOOK1  SYDE2  NEFL  NUDT12  SLC40A1  DCBLD2  RBM11  TRBV6-3  TPRG1  CLECL1P  RASGRP2  GJB6  H4C6  TNFSF11  IL9  EGLN3  AREG  CALD1  TRBV28  ADGRA3  PCLAF  B4GAT1  IL22  CDK5R1  LOC102723996  MPZL2  CRTAM  KCNK1  IL16  ANKRD18A  LOC107984120  PLA2G12A  NUDT11  GINS2  TGFBR3  TCEAL4  IL17F  UHRF1  LINC01806  SERPINB2  TRAV27  TRBV12-4  NPTX1  ZNF726  NPHP1  XCL2  IL17D  ALDH5A1  SCARNA10  ANKEF1  SNORD96A  KCNQ5  TRAV13-2  BEX3  PARP2  FAM169A  OIP5  TRBV12-3  SCARNA12  EXO1  SLC16A10  SCARB1  ZNF367  SCARNA5  ADAM23  SHISA2  ARMCX1  CORO6  IL17A  CENPV  XCL1  LOC105373204  ASB13  ZFYVE9  SCML2  DHFR  MATK  LRP6  PPP4R4  NDRG4  IL4  SNHG5  GPR63  KBTBD11  CMTM8  SATB1  NLRP2  MCM10  RAD54B  SCARNA28  LOC105379176  ORC1  DTL  BEX2  ISM1  HPDL  RFESD  CD69  ZNF595  TRAV16  MAD2L1  KIF15  XRCC2  TMEM170B  BBS5  TACC3  RPS28  ERCC6L  TRAV9-2  WWC2  PRXL2A  PRR7  HMGA1  KRT18  IL24  LOC107985023  DPY19L2P2  IFT22  SSX2IP  SNORD15B  RRAGD  GPRASP1  MFAP3L  LOC101927613  LY9  INSM1  TTK  TRAV23DV6  TRAV14DV4  SPRY1  DDIAS  IL2  NME4  JAM3  ZC4H2  RNF130  TTC8  POLE2  SDHAF3  SCARNA6  RASA4  KNSTRN  ZNF165  GNPDA2  SNORD87  MCM4  CHEK1  TRABD2A  CDC25A  TMEM71  RASA4B  SNHG6  CDC45  ZNF154  RPL21  RPL34  SNORD133  ORC6  CCR4  CDC20  GPD1L  HILPDA  SNORD110  MMAB  PLK4  E2F5  RGMB  ICA1L  PCNA  PPIL3  LOC112268121  MEST  GTSF1  PALS2  ZWINT  SORD  MCMDC2  MTIF2  DUT  IGSF3  LOC107984667  TRAV13-1  SMIM11  AKAP7  LTA4H  CENPH  EML5  SNORD101  C9orf40  EVI5  CCND3  SEC61G  FAM104B  HSPE1  ITGA6  FAM117B  HASPIN  RPS21  LOC105372310  MPP7  LYRM7  LDHB  LIAS  TMOD2  CETN3  TRBV5-1  SNHG21  SECISBP2L  ARL6IP5  WHAMM  PPM1K  RAB29  LDAF1  PTK2B  NPIPB6  RAPGEF2  APOBEC3G  TRIM25  LMO4  PLCXD2  CASS4  RCAN2  C3orf20  WARS1  FTH1  LOC107984200  CES4A  LGMN  CPEB3  TBC1D13  CLIC5  PDE4DIPP2  PARP4  SAMSN1  TNFRSF11A  GADD45A  LINC00342  F2RL2  LOC105369326  APOBEC3D  SPDYE6  HS3ST3B1  IRF9  XRN1  NPIPB13  ANXA5  EVI2B  INPP1  TRIM56  ZNF438  LOC101928214  SESTD1  TTC38  ERGIC1  NFKBIA  KAT2B  BEST1  LOC105369663  HLA-C  XYLT1  LOC646471  LOC101927723  BMERB1  CHST11  KLHL24  VANGL1  ARRB1  TSHZ3  LOC112268267  ZNF75D  PTPRJ  RELL1  SFMBT2  ZNF831  SOCS2  CPPED1  SHFL  SPDYE2  SRGAP2D  WIPI1  ZC2HC1A  LOC374443  HDAC9  YEATS2-AS1  OPN3  FAM174B  CTNND1  ERCC6L2-AS1  NPIPB3  CTSO  BCL2L1-AS1  LACC1  NPIPB4  CREG1  SNX11  HECTD2  MAP3K20  FGD6  PABIR3  NIPSNAP3A  NPC2  GVINP1  GLIPR2  MMD  CD4  RASGRP3  PLEK  LATS2  IPCEF1  LBH  PLEKHG1  ARHGAP15-AS1  MIR7848  LOC105372877  OPTN  VIM  TAP1  FRMD4A  SAP30L-AS1  IRF8  LOC105374407  RGS3  DNAAF9  LGALS3  PIK3C2B  LOC107984993  PRDM8  NEK10  CHRNA10  SCUBE3  CAPN2  ZNF189  TNFSF4  SQSTM1  LOC100130744  ZNF528  LOC101928512  LINC01160  CCDC141  IL12RB2  SPDYE2B  HPGD  TP53INP2  SNX9-AS1  CALHM2  SFXN3  TBC1D8  C3AR1  IGLV5-52  N4BP1  ATF3  TRAFD1  LDLRAD4  ANXA2P2  MOSPD2  FBXO32  LOC105373802  KMO  MYO1C  KIAA0040  LACTB  ANTXR2  IRF1  APOL2  LOC107986437  DLGAP1-AS1  PRAG1  SH3PXD2A  MYOF  TENT5A  BST2  NIPAL2  PSEN2  GSAP  BTN3A1  ARHGAP10  SRGAP2  DUSP5  TIAM2  CIITA  LOC105375785  SLCO4A1-AS2  LOC107986707  DNAH17  RDH10-AS1  MREG  KDM7A-DT  SDHAP1  AUTS2  CCDC136  LOC105374838  SPATA1  RASGRP4  OTUD1  TBX21  SLC41A2  JMY  IFNGR2  ATP10D  PSD3  JHY  DLG5  LOC105377291  ZBTB38  ANXA2  CD274  APOL6  ATP8A1  SOCAR  OAS2  LOC107986364  SLC25A53  SMARCA2  TP53INP1  IKZF4  LOC112268100  NUTM2E  ESR1  ALOX12  LOC644285  RRN3P3  FNDC3B  BMP2K  SOCS3  LOC107987043  CRADD  EFNB2  GCNT7  RIPOR3  TNFAIP3  ABL2  CPEB2  ATP8B4  NDRG1  NLGN3  BCAR3  MRTFA-AS1  PDE4DIP  SAT1  NPIPB5  LOC105373467  MINPP1  MRTFA  CTSS  SYNE2  FAS  TMEM140  BBS12  LOC729218  TUB  LOC107987147  SDHAP2  LOC105379395  TSPAN13  LOC107987234  LINC00102  CD84  F11R  DENND5A  DOCK4  PLAGL1  LRRC32  AKT1S1  NIPSNAP3B  STAT2  ADA  TRIM16  ADGRE2  C1orf21  SPRED1  LHFPL6  FAS-AS1  LRGUK  LSP1  TRIP10  HCG26  LOC105372064  CDYL2  GBP3  TRIM22  RRAGC  RBM47  MEAK7  EDARADD  P2RX4  ADCY9  LOC105369968  DISC1  LGALS3BP  HDX  H1-0  NRP1  LOC107984747  LOC105378841  ADORA2A-AS1  IFITM1  CALCOCO1  FGGY  LAG3  AHNAK  PSMB9  S100A11  IL6R  MAP3K20-AS1  ZFP36  SNX24  CFB  SLC35E3  H6PD  LOC105375945  SHE  TOX-DT  KIAA0825  CD68  CYP1B1  IRF5  LOC100507412  GAB2  LOC105374298  LGALS9  FBXW4P1  LOC642361  TANC1  AMPD3  LRP12  PMEPA1  LOC105372105  PDGFA  HECW2  HSH2D  ACAN  NINJ2  STIMATE-MUSTN1  PLB1  LOC112268458  C2CD4A  SLC9A9  IL36RN  RAB23  CCL1  SOCS6  APOL1  SLC22A23  RUFY3  CST7  IL18RAP  BIRC3  TUBB6  SLC15A4  FCHO2  LOC101928152  AFTPH-DT  YBX3  BTBD3  SDC4  TRANK1  LOC102723539  CCR1  LOC107984275  TPP1  FCHSD2  ATP8B1  TOGARAM2  SLAMF8  IL3RA  C4orf19  SLC43A2  CREBRF  ATP1B1  CARD6  FGL2  PTPN12  PIK3R5  NEDD4L  ITGA5  GOLIM4  LOC107987293  LOC105369315  NEDD9  CD83  MICAL1  VAV2  LOC105370504  LOC105372651  SPATS2L  CCRL2  RIGI  LOC101927741  MSC  DENND2B  RGS6  PLD2  FADS3  ARHGAP42  BTN2A2  RGL1  CXCL2  GRINA  ANGPTL7  RALB  PHLDB1  STIMATE  ANKRD10  ITPRIPL2  MTSS1  LOC107985120  INKA2  LOC102723458  MIR6891  SERPINB9  BISPR  ADPRH  GREM1  LOC105371932  STARD7-AS1  CXCR6  SHC4  SRGAP3  OPRM1  LOC107986604  RIMS3  KLF7  HAPLN3  LOC105379010  IFIT2  DDX60L  EHD4-AS1  ALPK1  FCGR2C  SRSF12  RAB5B  SUOX  LOC105378925  LOC112268426  LOC112268296  MYH11  LOC107987294  PARP14  CLIC4  ATP2B4  PLSCR1  NAV2  MIAT  SERPINB9-AS1  MOV10  MYLK  LOC105377663  LOC107985193  LOC105377296  SORBS1  UCP2  IFIH1  GNB4  PML  MBOAT2  LOC101927971  GM2A  GBP4  WNT2B  NINJ2-AS1  CHST3  YPEL5  TSPAN33  DPYSL2  TNFSF9  HOMER2  ABCA10  IL10RA  LHFPL2  FRG2B  LOC105378833  LITAF  CTSZ  F2R  SOX5  TMCC3  PAPSS2  GBP5  GLRX  FCGR2A  ZFHX2  CMYA5  CD163L1  JUN  C10orf67  PPP2R2B  MUC12  TRPS1  ABTB2  NABP1-OT1  DTX3L  FNIP2  CXCR4  LOC107984312  SMIM35  IFI35  NTRK3  ACTA2-AS1  EAF2  RSAD2  PLXNA2  VSIG10  HYDIN  LRRTM2  EFR3B  PARD3B  IFI30  GRIN2B  N4BP2L1  THBS1  SMIM14  ZNF618  DSE  EVC2  CDC27P11  CCR5  SMIM38  C1RL  GPR132  SLC15A2  ARHGAP31  TDRD7  DENND3  FBXO6  KCNQ3  TLCD5  GCNT1  A2M  SLC31A2  CARD16  LRFN2  LOC105374121  TAGLN3  TMEM150C  FRK  HIP1  MARCKSL1  LOC112268022  PNRC1  IL1B  GBP1P1  EREG  LOC102724621  LINC02641  AQP9  SMTNL1  CDC27P9  LOC105378228  GUCY1B1  P2RY1  RIPK2  CDC27P10  ITGAX  RAPGEF3  PARP12  ADAM19  FCER1G  TMEM200A  VWA5A  TPST1  OASL  TYMP  EHD1  FANK1  STXBP5L  CD34  PERP  CCR7  HSD11B1  ALPK2  MX2  PLAUR  ST3GAL5  LOC105376090  LOC107984301  GRB10  SAMD9  DUSP10  STAMBPL1  PLAT  MUC16  PTMS  UCP3  IKZF2  PARP9  MAFF  LOC105373342  DDX60  AKAP5  MUC6  LINC00158  HLA-DQB1  COL5A3  CDK14  IFIT1  CD38  BCL3  TOX  SSTR2  MAPK4  CREB5  SEMA4C  BCL2L14  ISG20  RASL11A  LOC105370461  ZFHX3  WAKMAR2  LINC02940  HMOX1  RTP4  MUC3A  DAPP1  IL31RA  C4orf50  KIAA1217  HAVCR2  GPRC5A  NTNG2  IL7  PPARG  PLA2G4C  CASZ1  GBP1  LOC105375914  CHST15  TRIM36  CCNG2  B4GALNT1  CCDC50  P2RX7  CDCP1  KCNMA1  LOC107985980  NIBAN1  ITIH4  DAB2  WHRN  COL9A2  GPR19  FCRL3  HLF  RASGEF1B  LOC105374071  IL1RN  CD74  ANKRD33B  CCL17  SMAD7  CTSK  TPPP  EPSTI1  CAPG  DNAH12  HLA-DQB1-AS1  TOR4A  TBC1D4  CMPK2  LINC02605  TUBB2A  FOXP3  CCR8  CBX4  CEACAM1  FGR  TUBA8  EVC  GPR137B  RHOC  DST  ADGRG1  HELZ2  SOD2  FMNL2  CDKN2B  MDGA1  BCL2L2  ATP9A  CSF3  RNF19B  LOC105372945  ODAPH  DRAM1  ACTA2  IRF7  G0S2  TFEC  LYN  DENND3-AS1  CD9  SAMD12  TENM2  TCF4  IFI6  PTGIS  ADRA1A  TUBB2B  MX1  TFPI  NRCAM  MMP14  ADAMTS4  E2F7  CRYBB3  RGS1  SPATA6L  LOC105372165  IGFN1  HMSD  SLC7A11-AS1  TSPAN2  SLAMF7  DOCK5  TNFSF13B  RUBCNL  TJP2  SERPINB9P1  ZC3H12C  LDLRAD3  POPDC2  LOC105370572  XIRP1  ART3  KIF13A  APOBEC3H  CXCL8  TTYH2  IRAK2  DHX58  LILRB4  MUC4  CCNA1  ZNF233  LOC653513  ITGAM  CD70  DAPK2  SH3TC2  IGFL2  CLCF1  COLGALT2  LOC105370363  MVB12B  FSD1L  OAS1  PDGFB  KIAA1671  ETV7  SRGAP1  DUSP1  NUB1  LOC105375913  FAM171B  ENOX1  SPRED2  LOC105374809  HLA-DPA1  SLC7A11  SLC22A1  TMCC2  CTTNBP2NL  XAF1  SAMD9L  MAOA  HLA-DPB1  TTBK1  ANPEP  RASAL2  LOC102725082  INHBA  SNN  CD93  SWAP70  PLAU  OAS3  SLC2A6  POU2AF1  OVOS2  TFPI2  PDCD1LG2  LAT2  SGMS2  ABLIM3  LMCD1  HLA-DRB5  ICAM1  GLDC  NAV1  SERPINB1  HLA-DOB  BMAL2  NUAK2  CDKN1A  GFPT2  USP41  LAYN  OXTR  VWA5B1  FBN1  CSF2RB  SYNM  USP18  RNF144B  SHTN1  MIR147B  DSC2  PRRG4  SRC  HLA-DMA  ALDH1A2  WDR86  RASSF8  LIPM  LAMC1  CKB  LOC105372647  PVR  SLC8A1  LOC107986589  HLA-DOA  ELOVL7  WWTR1  HLA-DRB1  ROR1  CCL22  DMD  MYO1E  CNTLN  ANKRD22  PLS3  CXCL16  LINC01181  SERPINA1  C15orf48  MIR3945HG  ITGA9  LOC105374062  VCAN  LINC02694  LAMP3  LINC02099  EBI3  GPR55  NRP2  CYRIA  GPR157  CXCL10  MACROH2A2  SEMA3C  PARM1  BMF  EPHA2  PLXND1  IL13RA1  DOCK1  ICA1  ADAMDEC1  IFI27  HLA-DRA  TNS3  GAS6  LOC105371356  HLA-DQA1  CD80  RAB31  WDFY4  MGLL  HLA-DRB6  DAPK1  NPFFR1  HLA-DQA2  STAC  SYNPO2  GJA1  ITGB8  EPB41L3  FGD2  SLC1A1  LRRK1  GSN  LOC107986127  KYNU  BASP1  SYNPO  CXCL9  CNKSR3  CXCL11  IL15  SLC8A3  SIRPA  BAALC  HCK  MMP7  MMP12  GCNT2  NEURL3  TGM2  SLC1A3  CYP7B1  LINC01050  CD40  LYZ  UBD  SVEP1  ENTHD1  CD86  NECTIN2  MARCKS  SERPINB7  SLC1A2  TBC1D9  IDO1  CRLF2  SLC6A12  LINC02244  CSF2RA  FLT3  LAMB3  EHF  CDC42EP4  ROR1-AS1  CLIC2  JAG1  GJA4  CXCL13  RASSF4  LIMCH1  FSCN1  TNFAIP2  ANXA3  DLL4  LINC03025  LAD1  IL27  LOC101929800 | DEFA3  S100A12  SFRS16  ALAS2  GLRX5  ERAF  HNRPA1P6  ALB  S100A9  PI4KA  PRKD2  HVCN1  SLC27A3  MAOB  EPR1  HNRPA3  CTA-221G9.4  CHPT1  CST7  CDC34  KLF4  MYB  CHST12  TOR3A  TCL1A  VNN2  VIT  ATP2C1  PHTF1  ELL  SHOX2  KIAA0515  OR52A1  UBE2V2  ORF1  FAM32A  C12orf32  PGPEP1  PUM1  PDAP1  ZC3H7B  HNRPD  CCNDBP1  TNRC6C  HLA-DRA  PUNC  FAIM3  B2M  G6PC3  CPEB3  SNRP70  ACTB  VSIG2  CSTA  ATIC  SNX3  NUDC  SLC25A24  AZI1  TXN  NDUFB1  SYT11  CENTD2  SLC25A37  C1orf56  C15orf49  DARC  BTN2A3  LDHC  LGR6  TNFRSF10A  B3GNT2  TDRD1  ACTG1  VCP  FRS3  CD79B  IRAK3  B2M  EXOC7  INTS1  PDZD8  SEBOX  AURKC  ATP5E  HNRNPU  EIF3EIP  B2M  RBP4  SFRS5  BOK  LY9  B2M  PPBP  MN1  LCOR  CD37  MCM5  PHF17  FAM57B  EGR4  JUND  REC8  FNIP1  B2M  NUDT1  SP140  MFN1  SERHL2  MLYCD  RPS15A  LAT  MAPK8IP2  C8G  CA1  KAL1  B2M  B2M  TRIM25  LGALS3  PARVA  C1orf43  KARS  ZNF12  SIAH2  UBE2I  ACTB  BAIAP2L2  HNRPK  TTLL3  TNK2  SLC40A1  SAPS2  CLC  RENBP  B2M  ALG9  B2M  HEBP2  LMNB2  BNIP3L  TRAP1  MAP4  PTK6  CACNA2D3  CAPRIN1  FRYL  LRRN3  CCDC85A  ZNF714  B2M  C13orf7  FAHD1  TUBGCP2  GPX3  GPHN  GTDC1  ARR3  HNRPM  CHD9  TPCN2  ESR1  KCNH2  SLTM  EPHA3  B2M  FARSB  PDIA3  B2M  B2M  HLA-DQB1  HLA-DPA1  GSR  DCTN3  TMEFF2  IVL  LIMD1  B2M  B2M  NCDN  BCL2L14  AES  KLK1  TXN  HLA-DPB1  ND6///ND4L///ND3///COX2  ARHGAP1  PPM1A  CNIH4  RNF10  B2M  FOXK2  ACTN1  C20orf117  MX2  UQCRQ  B2M  IFIT1  SUPT7L  SRGN  C14orf159  EFHC2  RAB24  SPG7  UBE1  KCNQ2  ALS2CR2  SLC25A37  MRPS7  FAM3A  IL1F5  GIMAP4  C14orf2  ECH1  SAMSN1  C21orf105  BLVRB  CDA  PSME3  RPL21  SERPINC1  GNMT  IL1R2  GPR18  NDUFB3  RPL39L  TMEM85  STAMBPL1  GOLGA2L1  B2M  HUMBINDC  B2M  UROD  HNRNPA2B1  KARS  BCL2A1  RPS3A  EPB42  ACTB  ALPL  AMD1  GMPR  GAPDH  DMAP1  USMG5  ENO1  RPS7  LCK  B2M  ANXA6  MS4A7  ZNF160  HNRNPA1  CEACAM6  GZMA  CCT3  CYFIP2  C11orf2  CEACAM4  ARG1  CIDEB  KIAA1147  METTL9  B2M  FCGR1A  POLR3C  USPL1  RNF213  WARS  GAPDH  FCER1G  IL32  ACTB  ATP6V1C1  GAPDH  RPL17  C4A  IFIT3  CCDC42///IFI6  ANXA2  RPS29  RBMX  GABARAPL2  GAPDH  ACTB  C4orf8  GRID2  EIF4A2  S100A11  PSMB9  IFIH1  ACTB  SPATA2  GCA  HERC5  ANXA1  RPL9  OASL  RPL28  TRDC  CAMP  C1orf217  RPS17  FPRL1  CLN3  RPL34  ISG15  UBL5  LDHB  PBEF1  RPL31  IGJ  RPS21  ACSL1  RPL27  RPL7  TXNIP  RPL23  C9orf86 | CNR1  CHRNA2  CHRNB3  CYP2C8  SLC47A2  ABCB5  ABCB1  STIP1  CYB5R3  PYGL  DPYD  CYB5R1  CES1  VWF  IL4I1  FABP1  GLS2  RARRES1  PRKCI  CYP2C19  PRDX6  PC  GPRC5A  MMP12  ARG1  SLC22A7  CSTB  BLMH  CPT1A  SLC2A2  SULT2A1  INS  RBP4  ALPL  GFER  TNFAIP6  FBP1  ABCC6  GPT  ABCG5  ABCG8  CYP1A2  HNF4A  CYP1A1  CYP2C18  GPX7  CYP4B1  PRODH  NR1H3  CYP3A5  CYP3A7  SLCO1B3  APOA1  TTPA  WARS  SLCO1B1  HSPG2  CYP2B6  S1PR1  APOL1  FDXR  PLD2  MCL1  EIF2AK2  CARTPT  AKR1C1  CXCL10  RETSAT  ITIH4  BLVRB  TXNRD1  CYP2C9  ADRA2C  COX8A  PSG5  SGPL1  NPC1L1  NR4A3  NDUFA13  CDK15  NR1H2  PFKFB1  ADRB3  SEPSECS  SIK2  GLO1  ITGB2  STAT5B  GCK  BAAT  FABP6  ADRB2  ADRB1  ADSS  CD38  CYP3A4  TNFSF11  PDCD6  SERPINB3  PKLR  KDR  FFAR4  RHO  CD80  PRKCE  ALDH3B  PTPRE  HRH3  EPOR  EGLN2  ADI1  MAPK14  PIK3R1  PIK3R2  NOS3  ADRA2A  ADRA2B  FLT3  CCKAR  GRB2  PRDX5  TNFRSF1B  CES2  RYR3  RFK  S100A13  ROCK1  ASIC1  PTPN1  ITPR1  HNRNPH3  PTGDR2  ANG  PRKCD  PARK7  CAMK1D  SERPINA1  HSP90AA1  NEK1  TRPM7  CD33  CIB2  RARA  RPS6KA4  AURKC  CYP1B1  KCNB1  PRKCA  A2M  PPBP  NISCH  MAP2K5  PRKCB  HSPA8  RHOB  EPRS  GDF5  CD22  RAC1  DGAT2  HCAR2  GPER1  CD44  PPIF  NRP1  PSMB8  MYLK  PDGFRB  CCN3  TLK2  RANBP1  EPHB6  CXCR4  PRKCG  KNG1  PDPK1  RPS6KA3  LIMK1  TAP1  ROCK2  PKN2  TEC  PAK6  TLR4  NUAK2  STK24  GAPDH  TRPM8  EPHA2  TNK2  PRKCQ  PRKD1  IKBKB  NOD2  MAPK13  PRKAA1  DAPK3  SIK1  EGLN3  ACVR1B  FYN  KCNA2  CIB1  PTK6  PDGFRA  MAPK9  TYRO3  PZP  JAK2  BMX  AURKB  RPS6KA1  PRKACA  PIK3CB  RORA  NUAK1  KCNQ1  MAPK7  MKNK2  MAP2K6  GSK3A  PIK3CD  JUN  CPNE1  MAPK1  FES  CDK2  NFKB1  PML  ARL2  ABL1  ULK2  EPHA4  MAPK11  CLU  STK3  HIPK2  MAPKAPK5  EIF6  PAK2  CHP1  TRPV4  AKT1  IKBKE  PAK1  SFPQ  S100A9  CYP3A43  PRKCZ  ADRA1A  ADRA1B  ADRA1D  ITPR2  ITPR3  CYBB  CYBA  NCF1  NCF2  NCF4  RAC2  PRKAA2  PRKAB1  PRKAB2  PRKAG1  PRKAG2  PRKAG3  NFKB2  TNF  IL6  TP53  IL1B  IFNG  F2  IL10  TGFB1  FAS  CASP3  ALB  NOS2  CASP8  EGFR  CXCL8  ICAM1  CYP2D6  FASLG  PTGS2  CCL2  VEGFA  APOE  HLA-DRB1  ACE  MYC  HLA-B  MPO  CTNNB1  PPARG  HIF1A  CD40LG  CYP2E1  IL2RA  CCND1  BCL2  F5  HFE  IL2  MAPK8  SLC17A5  LEP  CTLA4  HMOX1  SERPINE1  EGF  MMP9  PTEN  TNFRSF1A  CRP  TF  MTOR  SLC2A1  NR1H4  HLA-A  CAT  NGF  GFAP  HGF  THBD  F3  ADIPOQ  CPT2  SOD1  IL1RN  FOS  IL4  CDKN2A  CCL3  IGF1  EPO  STAT3  IL13  IL1A  APOB  IFNA1;  EDN1  PIK3CA  COMT  BDNF  RYR1  UGT1A1  DRD2  BAX  NLRP3  REN  G6PD  CYCS  APP  CD36  KRAS  KIT  CCR5  ERBB2  XIAP  CXCL12  CFTR  TNFSF10  MAPT  PPARA  NAT2  THPO  MAPK3  NFE2L2  TERT  HLA-DQB1  F9  ABCB11  BCL2L1  BMP6  MET  CALCA  HRAS  APC  GSTM1  LPL  CASP9  IL5  SNCA  ABCA1  FGF2  ENG  IL18  CSF3  SERPINC1  HADHA  CALR  RELA  GGT1  ITGAM  AFP  CDH1  TTR  LDLR  ELANE  PARP1  CDKN1A  F8  ABCB4  TH  TYMS  BMP2  TLR3  GSTP1  STAT1  ABCC2  AGTR1  HLA-DQA1  SELP  HMGCR  INSR  IGF2  SPP1  CSF2  DMD  F10  CCL5  PDGFB  CDK4  IDH1  HPRT1  IL3  PTPN22  FCGR2A  POLG  SST  CCL11  C4A  LMNA  FOXP3  TPMT  IL17A  SELE  C3  BRCA2  VCAM1  ADAMTS13  MMP2  CHAT  LRP5  SOD2  MEFV  ESR1  ELN  TLR2  MMP1  IDH2  HAMP  MBL2  SLC25A13  ENO2  NQO1  PTPN11  S100B  HP  IFNL3  ACHE  CAV1  PTGS1  HNF1A  BIRC5  ATM  PLG  PRKN  SPTAN1  SCN1A  C4B  MTHFR  PRL  HLA-C  AGT  KRT8  CHKB  CREB1  CDKN3  G6PC  MECP2  MT-CO1  GAA  XDH  GATA1  VIM  PTPRC  ATP8B1  UCHL1  NPPA  KRT18  PCNA  POMC  ANXA5  PRRT2  PLAU  CD79A  ABCC1  ABCG2  COX5A  NR1I2  CD40  HSPA4  PLAT  BGLAP  NRAS  LEPR  FADD  PDCD1  TKT  SRC  CYP2A6  NDUFS4  PIK3C2A  KITLG  GDNF  HTR2A  IFIH1  IRF3  IL1R1  BCHE  TFRC  CEBPA  BRAF  NTRK1  HMGB1  GSR  ALOX5  FGFR2  PIK3CG  CSF1  ALPP  SERPINA3  IRF5  CD81  NPY  TNFRSF10A  VDR  NTRK2  CASP1  CXCR3  HLA-G  NOTCH1  MAOA  PRF1  PNPLA3  HBB  F7  CASK  SETD2  CFH  DHFR  TIMP1  ATP7B  MIF  ITGB3  RAF1  B2M  NR3C1  CP  CRH  NOS1  NBN  MSR1  FAH  ABCC8  PPIG  ADA  TNFRSF10B  GRIN2B  FGFR1  BRCA1  MDM2  OPRM1  IFNB1  WT1  RETN  TG  MUC1  TRPV1 |

Supplementary Figure S1. Funnel plot of publication bias for the overall effective rate.

Supplementary Figure S2. Funnel plot of publication bias for c-reactive protein (CRP).

Supplementary Figure S3. Funnel plot of publication bias for erythrocyte sedimentation rate (ESR).
